# Adjustment in the Composition and Organization of *Proteus mirabilis* Lipids during the Swarming Process

**DOI:** 10.3390/ijms242216461

**Published:** 2023-11-17

**Authors:** Paulina Stolarek, Przemysław Bernat, Antoni Różalski

**Affiliations:** 1 Department of Biology of Bacteria, Faculty of Biology and Environmental Protection, University of Lodz, Banacha 12/16, 90-237 Lodz, Poland; antoni.rozalski@biol.uni.lodz.pl; 2 Department of Industrial Microbiology and Biotechnology, Faculty of Biology and Environmental Protection, University of Lodz, Banacha 12/16, 90-237 Lodz, Poland; przemyslaw.bernat@biol.uni.lodz.pl

**Keywords:** *Proteus mirabilis*, swarmer cells, rods morphology, phospholipids, fatty acids, lipid rafts

## Abstract

*Proteus mirabilis*, an opportunistic pathogen of the urinary tract, is known for its dimorphism and mobility. A connection of lipid alterations, induced by the rods elongation process, with enhanced pathogenicity of long-form morphotype for the development of urinary tract infections, seems highly probable. Therefore, research on the adjustment in the composition and organization of *P. mirabilis* lipids forming elongated rods was undertaken. The analyses performed using the ultra-high performance liquid chromatography with tandem mass spectrometry showed that drastic modifications in the morphology of *P. mirabilis* rods that occur during the swarming process are directly related to deprivation of the long-form cells of PE 33:1 and PG 31:2 and their enrichment with PE 32:1, PE 34:1, PE 34:2, PG 30:2, PG 32:1, and PG 34:1. The analyses conducted by the gas chromatography-mass spectrometry showed negligible effects of the swarming process on fatty acids synthesis. However, the constant proportions between unsaturated and saturated fatty acids confirmed that phenotypic modifications in the *P. mirabilis* rods induced by motility were independent of the saturation of the phospholipid tails. The method of the Förster resonance energy transfer revealed the influence of the swarming process on the melting of ordered lipid rafts present in the short-form rods, corresponding to the homogeneity of lipid bilayers in the long-form rods of *P. mirabilis*. Confocal microscope photographs visualized strong Rhod-PE fluorescence of the whole area of swarmer cells, in contrast to weak membrane fluorescence of non-swarmer cells. It suggested an increased permeability of the *P. mirabilis* bilayers in long-form rods morphologically adapted to the swarming process. These studies clearly demonstrate that swarming motility regulates the lipid composition and organization in *P. mirabilis* rods.

## 1. Introduction

The dimorphic, motile, Gram-negative, and facultative anaerobic bacterium *Proteus mirabilis* is commonly found in soil and water, as well as in the normal human intestinal flora. This opportunistic pathogen has been known to cause serious infections in humans, although the vast majority of *P. mirabilis*-mediated infections occur in patients with anatomical or functional abnormalities of the urinary tract and long-term urinary catheterized patients. Urinary tract infections (UTIs) continue to be a global problem affecting approximately 150 million people each year [1].

*P. mirabilis* is frequently mentioned in terms of its swarming motility. The swarming phenomenon is a surface-induced differentiation process of short (1–2 µm) single cells with a few peripheral flagella into elongated (20–80 µm), hyperflagellated, multinucleated, and non-septate forms arranged in rafts, which enables rapid movement of the cell population across solid surfaces in a highly coordinated manner. *P. mirabilis* motility is possible on both biotic (i.e., urethra epithelium) and abiotic surfaces (i.e., catheters, intravenous lines, and other medical equipment) [2]. Research proves that uropathogens are able to migrate over all-silicone catheters, silicone-coated latex catheters, and hydrogel-coated latex catheters [3]. It has been shown that cell migration across urethral catheters enables *P. mirabilis* swarmers to gain entry to the mouse urinary tract [4]. Moreover, it has been suspected that elongated and hyperflagellated cells could be well suited in an ascending route of infection in non-catheterized patients. Allison et al. [5] demonstrated the ability of *P. mirabilis* swarmer cells to establish the ascending infection of the mouse kidney. On the other hand, more recent studies have shown that in mouse models of ascending urinary tract infection, short rods of uropathogens dominate instead of long ones [6], which undermined the crucial role of swarming motility in ascending UTIs and pyelonephritis. Significance of swarming in the pathogenesis of *P. mirabilis* still remains to be assessed.

As mentioned before, motility is an important virulence factor because it could allow for the microorganisms to reach the site of colonization and then spread to other areas of the urinary tract. It has been documented that non-swarming or weakly swarming strains of *P. mirabilis* are characterized by a reduced ability of cell adhesion to the bladder epithelial cell line 5637 [7], weakened capacity to colonize the mice bladder and kidneys [8], and limited ability of form biofilm on abiotic surfaces [9]. Moreover, structural changes occurring in the rods during the swarming process resulting in a modified cell phenotype undoubtedly influence other aspects of *P. mirabilis* pathogenesis. It has been documented that swarmer cells demonstrate higher activity of urease, IgA metaloprotease ZapA and HpmA hemolysin, and intensified flagellin synthesis, in comparison to swimmer cells [2,10]. Interestingly, recently published research indicates that motile cells of *P. mirabilis* induce low levels of proinflammatory cytokines IL-6, IL-8, and IL-1β, as well as the antimicrobial peptide HBD-2, while stimulating high concentrations of the anti-inflammatory cytokine TGF-β in infected epithelial cells [9]. These morphotypes of *P. mirabilis* rods upregulate the intrinsic pathway factors, especially BCL-2, recognized as an anti-apoptotic factor which indicates the lowest degree of induced apoptosis in human prostatic adenocarcinoma PC-3 [11]. Taking into account the above, it seems reasonable to assume that the elongated cell type plays an important role in some stages of the bacterial infection.

Initiation of the *P. mirabilis* rods differentiation has been quite well recognized. The process requires inhibition of flagellar rotation plus accumulation of extracellular putrescine and O-antigen interactions with a surface [12]. It could be a response to specific nutrients, as well as environmental signals, and is regulated by quorum sensing [13]. It has been documented that proteins for flagellar biosynthesis and rotation, surface elongation, control and coordination of multicellular motility, lipopolysaccharide (LPS) and peptidoglycan production, and cell division are needed for successful transformation of swimmer cells to swarmer cells [2]. Studies conducted at the transcriptome level showed that during drastic morphological changes occurring in the bacterial cells, 541 genes were upregulated in short-form cells and only 9 genes were upregulated in long-form cells. Genes involved in processes such as flagellar biosynthesis, amino acid import and metabolism, oligopeptide transport, and cell division were upregulated in short-form rods [14]. The authors suggest that during the consolidation phase, *P. mirabilis* prepares for the next wave of swarming. A study which was focused on changes in peptidoglycan composition occurring during cell differentiation demonstrated that the level of peptidoglycan O-acetylation was lowering from 51% to 29% in the process of *P. mirabilis* rods elongation. It was accompanied by a simultaneous rise in the content of anhydromuropeptides and a reduction in the amount of Lys-Lys-muropeptides arising from bound lipoprotein. The latter pointed to the shortening of the muropeptides chain length in swarmer cells [15]. Interestingly, to understand biological systems where the spatial dynamics depend on the local physiological structure, Ayati et al. [16] developed composite hyperbolic-parabolic partial differential equations to model *P. mirabilis* swarm colony expansion. Despite the fact that swarming motility of this urinary tract pathogen has been extensively studied, modifications in the lipidome of *P. mirabilis* have been pushed to the background. In a paper published in the late 1970s, similarities rather than differences in the phospholipid composition between swimming and swarming cells were presented [17]. On the other hand, more current data indicate significant quantitative changes in the amounts of fatty acids (FAs) (lipids components) [18] and phospholipids (PLs) in the elongated rods of *P. mirabilis* [19]. However, unique spectral signals produced by infrared microspectroscopy were unable to specify the alterations in the bacterial phospholipid composition [19].

Cell membranes, composed of thousands of different lipid molecules, are dynamically responsive and form stable or transient structures which may be used by various proteins as platforms for their activity [20]. A vast majority of amphiphilic lipids located in membranes are glycerophospholipids, composed of a glycerol molecule, a phosphate group, two fatty acids, and a variable head group such as choline, glycerol, ethanolamine, serine, or inositol [21]. Phospholipids are recognized as factors of membrane-protein topology, required for proper transport of solutions and electrons, the initiation of DNA replication, and cell division [22]. Fatty acids, the acyl constituents of phospholipids, are listed among the most important cellular components. Alongside their structural functions, fatty acids serve as storage materials and energy suppliers in cells, and their derivatives are involved in cell signaling. For instance, *cis*-2-unsaturated fatty acids with different chain lengths and branching patterns are cell-to-cell signals which modulate bacterial activities in response to environmental conditions [23]. The adaptation to environmental conditions (sometimes extreme) allows bacteria to survive. This process may involve changes in their protein, sterol, hopanoid, and carotenoid contents proceeded by alterations in the composition of membrane lipids [24]. Modifications of the structure of glycerolipid acyl chains allow for maintaining passive permeability for hydrophobic molecules, active solute transport, and protein–protein interactions [24], minimizing energy expenditure and optimizing bacterial growth [22]. Considering the above, an assumption that phospholipids play an important role in the process of *P. mirabilis* cells differentiation seems to be proper.

Many bacterial lipid-associated virulence factors recognized by the human innate immune response, such as LPS in Gram-negative bacteria, lipoteichoic acid in Gram-positive bacteria, and lipoglycans in mycobacteria interact specifically with the host cell machinery during the bacterial infection. Despite the fact that both *Escherichia coli* and *Mycobacterium tuberculosis* differ in their cell envelope composition and organization, their cell wall-associated lipid components are considered as virulence factors disrupting host cellular signaling and finally leading to the development of diseases. A review of these lipid-derived molecules, active in various stages of host–pathogen interactions, was extensively presented by Dadhich and Kapoor [25]. Recently, information regarding aminoacyl phospholipids has appeared in the literature. These lipids have proven to significantly increase bacterial resistance to antibiotics, bacteriocins, and host defense molecules, as well as contributing to enhanced virulence. Moreover, membrane flexibility and stability regulated by aminoacyl phospholipids enables bacteria to control processes such as sporulation, conjugation, biofilm formation, and swarming motility [26]. On the other hand, lipid properties make them an attractive target for pathogens to modulate host cell processes to allow for their replication and survival. Various strategies which pathogens use for the modulation of cellular processes by modifying host cellular lipid homeostasis were summarized by van der Meer-Janssen [27]. Taking into account the connection between bacterial lipid factors and their cells pathogenicity, the recognition and understanding of the changes in the rod lipidome occurring during the swarming process are extremely important in the context of *P. mirabilis*-mediated UTIs development.

Biological membranes are composed of dynamic lipid and protein clusters, rather than forming a homogeneous mosaic. It has been hypothesized that affinity between lipids leads to the creation of lipid microdomains with unique compositions, which provide environments favorable to the activity of certain proteins. Recently, sphingolipid- and cardiolipin-enriched liquid-ordered domains drifting between liquid-disordered phospholipids, named as lipid rafts, are of particular interest due to their close relation with cell signaling proteins located both in the inner and outer membrane leaflets [20]. Lipid rafts or raft-like lipid heterogeneities containing high melting lipids may also determine and maintain the physical properties of membranes. This kind of domain has been noticed in some microorganisms such as *E. coli*, *Bacillus subtilis*, and *Staphylococcus aureus* [28,29].

The present study was focused on the adjustment in the lipid composition and organization in the *P. mirabilis* rods induced by the swarming process. Two uropathogenic strains capable of swarming motility were initially assessed for the duration of the lag phase, speed of expansion, and the visual appearance of the colony. Morphologically differentiated forms of bacterial cells were compared with each other in terms of their fatty acid and phospholipid profiles. This step was carried out by applying the gas or liquid chromatography separation of the cellular components and their identification by mass spectrometry. Moreover, short-form and elongated rods of the *P. mirabilis* were examined in search of co-existing ordered and disordered domains using the Förster resonance energy transfer (FRET). The research was undertaken due to the highly probable connection of lipid alterations induced by the elongation of *P. mirabilis* rods with enhanced pathogenicity of the morphotype developed by the swarmer cells in order to be more effective in causing UTIs.

## 2. Results

### 2.1. Expansion Rate, Behavior, and Visual Appearance of the P. mirabilis Swarm Colony 

**Figure 1:**
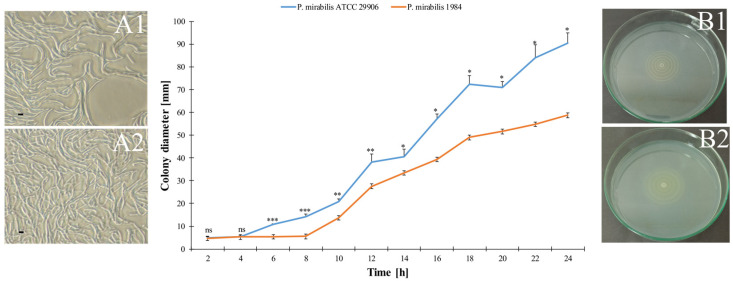
Swarming zones of the *P. mirabilis* measured over time. The error values equal the standard deviations. Statistical significance is indicated as * (*p*-value ≤ 0.05), ** (*p*-value ≤ 0.01), *** (*p*-value ≤ 0.001), and ns means not significant (*p*-value > 0.05). Panels A show images of the elongated *P. mirabilis* rods produced by phase-contrast microscopy (magnification = 600×, scale bar = 5 µm). Panels B show a characteristic swarming pattern, formed by *Proteus* sp. The ATCC 29906 (1) and 1984 (2) strains are presented in both panels.

Swarming motility of the elongated *P. mirabilis* rods (panels A1 and A2 in Figure 1) was quantified by a systematic measurement of the swarm colony sizes on an LB plate. Examined uropathogens differed significantly in the dynamics of swarming growth (a graph in Figure 1). The lag time for the 1984 strain took almost twofold longer than for the reference strain. As a result, the swarming zone formed in a motility plate by the *P. mirabilis* ATCC 29906 achieved 90.50 mm ± 4.50 mm after 24 h, while that formed by the 1984 strain reached 58.70 mm ± 0.56 mm. Based on this data, colony expansion rates were estimated at 3.77 mm h^−1^ and 2.45 mm h^−1^ for the ATCC 29906 and 1984 strains, respectively.

**Figure 2:**
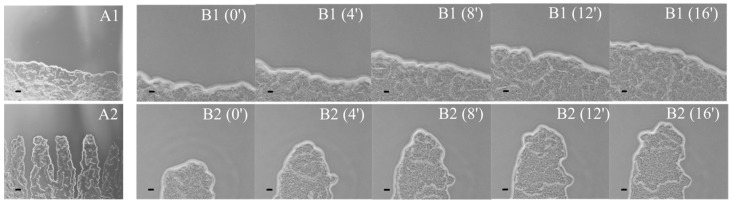
The time-dependent spreading of the ATCC 29906 (1) and 1984 (2) swarmer cells across an agar surface presented by a sequence of phase-contrast microscope images. Swarm fronts are shown in A panels (magnification = 40×; scale bar = 20 µm). Course of the edge of the swarm colony over a four-minute is shown in B panels (magnification = 200×; scale bar = 20 µm).

Macroscopically, bull’s-eye patterns formed by the tested strains appeared to be almost the same (panels B1 and B2 in Figure 1). However, greater colony spreading, as well as more visibly separated swarming and consolidation terraces, were characteristic for the pattern of the ATCC 29906 strain. Moreover, microscopic analyses showed relevant differences in the visual appearance of the *P. mirabilis* swarm fronts (Figure 2). Unlike the reference strain with a smooth front of the outermost migration ring, the 1984 strain was distinguished by forming finger-shaped projections in this plate region.

### 2.2. Alterations in the P. mirabilis Phospholipid Profile Occurring during Its Rods Elongation

The analyses performed using the ultra-high performance liquid chromatography with tandem mass spectrometry (UHPLC–MS/MS) provided important information on the qualitative and quantitative compositions of phospholipids building morphologically differentiated cells of the *P. mirabilis* formed during the swarming process.

Phosphatidylethanolamines (PEs) and phosphatidylglycerols (PGs) represented respectively almost 70% and nearly 30% of the total PLs content. The 26 major PL species identified in the bacterial cells are shown in Table 1.

A notable influence of the swarming process on the *P. mirabilis* lipid composition was indicated using our UHPLC–MS/MS generated profiles. Statistically significant changes in the levels of 23 PLs species, among the 26 identified, were found. The increase in the amounts of the 32- and 34-carbon phospholipids, as well as strong reduction in the levels of the 31- and 33-carbon lipids, were specific for all swarmer cells. The first one ranged from 36% to 600% for the PE 34:1 and PE 34:2 levels, respectively. The second was on the border of limit detection in most cases (like for PE 31:0, PE 33:0, PE 33:1, PG 31:2, and PG 33:1). It was confirmed that drastic modifications in the morphology of the *P. mirabilis* rods that occur during the swarming process were directly related to the changes in the phospholipid profile of the elongated cells. These alterations involved the deprivation of the long-form cells of PE 33:1 and PG 31:2 and their enrichment with PE 32:1, PE 34:1, PE 34:2, PG 30:2, PG 32:1, and PG 34:1, compared to the short-form cells. 

Interestingly, despite quantitative alterations in almost all PL species, the PG/PE and the double bond index (DBI) values remained unchanged (Table 1) after the elongation of the *P. mirabilis* cells. Constant PG/PE ratios resulted from compensating for the lower content of several lipid species with higher levels of the other species, within the same phospholipid class. For instance, the increase in the PE 32:1, PE 34:1, and PE 34:2 total quantity (~22.42%) was compensated for by the decrease in the PE 33:1 level (~23.23%), while the increased total content of PG 32:1 and PG 34:1 (~11.69%) was compensated for by the decreased total level of PG 31:2 and PG 33:1 (~12.14%). Therefore, proportions between PGs and PEs remained similar after the morphological modifications of the *P. mirabilis* 1984 rods.

The double bond index (Table 1), calculated for all PLs, referred to the average number of double bonds in the fatty acids esterified to polar lipid molecular species, indicating the level of PL unsaturation. Similarly to the PG/PE ratios, the DBI values remained constant for the *P. mirabilis* rods, independently of their morphotype. It was probably related to the negligible impact of the rods elongation process on the saturation of fatty acids, and a greater effect on their variable distribution, i.e., attachment to other phospholipid heads, leading to a formation of different PL species. These results suggested that the changes in the rods phenotype occurring during swarming motility did not concern the saturation of the phospholipid acyl chains of the *P. mirabilis* rods. 

**Figure 3:**
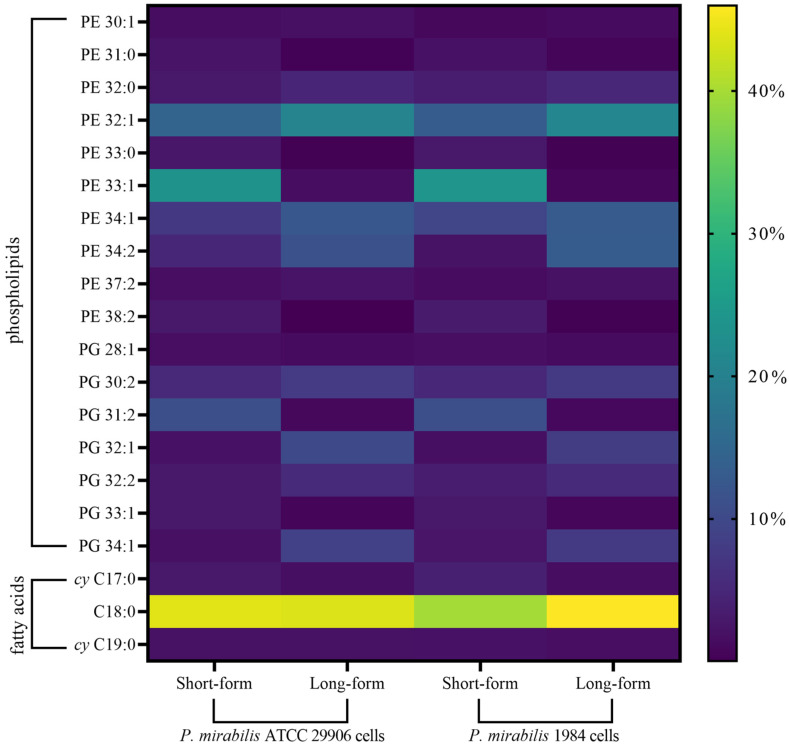
Heat map generated from quantitation of phospholipids and fatty acids in the short- and long-form rods of the *P. mirabilis*. Morphologically differentiated cells are shown on the lower *x*-axis, while the lipid analytes are presented on the *y*-axis. Only the seventeen most important phospholipids and three most important fatty acids (based on their levels ≥ 1% and *p*-values ≤ 0.05) are displayed. The features are color coded by row with yellow indicating high intensity and purple indicating low intensity. The underlying numerical data used to generate this figure (along with statistical significance values) are available in Table 1 and Table 2.

A visual summary of the above data is presented as a simple heat map in which colors represent the obtained values (Figure 3).

### 2.3. Modifications in the P. mirabilis Fatty Acid Profile Occurring during Its Rods Elongation

Using a gas chromatography-mass spectrometry technique (GC–MS), 12 types of fatty acids in the morphologically differentiated cells of *P. mirabilis* formed during the swarming growth were recognized and quantified. Mass spectra illustrated that acyl chains of FAs varied in length between 14 and 20 carbon atoms. The major saturated FAs (SFAs) were hexadecanoic (palmitic) acid (C16:0) and octadecanoic (stearic) acid (C18:0), while the major unsaturated FAs (UFAs) were 9-hexadecenoic (palmitoleic) acid (C16:1) and 9-octadecenoic (oleic) acid (C18:1), both in the *cis* conformation. Two cyclopropane FAs (CFAs) called 9,10-methyl hexadecanoic acid (*cy* C17:0) and 11,12-methyl octadecanoic acid (*cy* C19:0) were also identified, both in the *cis* conformation (Table 2). The obtained results show that the 15 PEs and 11 PGs of *P. mirabilis* were composed mostly of C16:0 and C18:0 (~80% in total), and to a lesser extent of C16:1 and C18:1 (~5% in total).

The swarming process affected FA composition of the 1984 strain more than that of ATCC 29906. As shown in Table 2, the levels of *cy* C17:0 and *cy* C19:0 were significantly decreased (by about 62% and 22%, respectively) in the long-form rods of *P. mirabilis*. In effect, the CFA/UFA ratio was reduced from 1.13 to 0.74. Interestingly, the saturation of fatty acyl chains increased significantly in the elongated 1984 cells, in contrast to the branched fatty acyl chains, which remained unchanged. On the other hand, the constant values of UFA/SFA for both *P. mirabilis* strains were in line with the unchanged DBI values presented in Table 1, which confirmed that phenotypic modifications in their rods induced by swarming motility were independent of the saturation of the phospholipid tails. A vast majority of other FA modifications occurring in bacterial cells during their movement turned out to be statistically insignificant. It seems that the swarming process has a negligible effect on fatty acids synthesis but may affect FA distribution for the formation of the phospholipid species required by the long-form rods. 

A visual summary of the above data is presented in Figure 3.

### 2.4. Lipid Organization in the P. mirabilis Rods

**Figure 4:**
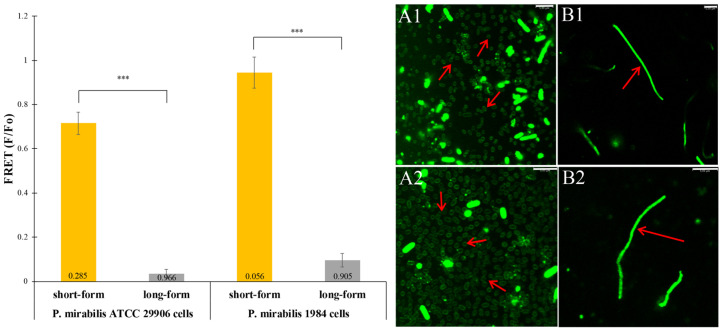
Ability to form ordered-state bilayers by the *P. mirabilis* lipids, as assayed by the Förster resonance energy transfer technique. The values of fractions of energy transfer are located at the bottom of the bars. The error bars equal the standard deviations. Statistical significance is indicated as *** (*p*-value ≤ 0.001). The confocal microscopy images present short- (**A**) and long-form cells (**B**) of the ATCC 29906 (1) and 1984 (2) strains stained with a membrane probe Rhod PE (magnification = 1000×; scale bar = 5 µm). Red arrows indicate morphologically differentiated bacterial rods of *P. mirabilis* labeled with the fluorescent probe.

The FRET method was used to reveal the degree of the bacterial membrane order. The donor (pyrene PE) was distributed into the liquid-ordered phase, while the acceptor (rhod PE) was located in the liquid-disordered phase [30]. The ratios of donor fluorescence in the presence of the acceptor to that in its absence (F/Fo) are presented in Figure 4. High F/Fo values (even 0.94 ± 0.07) were observed in the short-form cells of *P. mirabilis*. Weak FRET corresponded to strongly ordered bilayers. The swarming phenomenon led to a significant reduction in F/Fo values for the bacterial rods. High values of energy transfer fractions from the donor to the acceptor (even 0.966) corresponded to the homogeneous lipid bilayers of the elongated *P. mirabilis* rods.

The green channel of the confocal microscope visualized the Rhod PE-stained *P. mirabilis* membranes. The short-form rods displayed a weak membrane fluorescence, while the elongated rods showed a strong fluorescence of the whole cells (panels A1–B2 in Figure 4). The imaging confirmed modifications in the arrangement of the bacterial membrane components occurring during the swarming process.

## 3. Discussion

*P. mirabilis*, a member of the *Morganellaceae*, is a urinary tract pathogen which is problematic for catheterized patients. Complications include not only a catheter blockage by encrustation but also a risk of ascending infections enabled by uropathogen migration over the catheter surface. Nevertheless, the role of swarmer cell differentiation in the virulence of *P. mirabilis* is still a matter of debate.

In laboratory conditions, a *P. mirabilis* colony spreads on agar forming the bull’s-eye pattern by consecutive phases of rapid swarming followed by consolidation into shorter rods [14]. This characteristic pattern was observed during the migration of the tested uropathogens. However, the consolidation zones of the 1984 strain were tightly clustered, which meant shorter periods of swarming, compared to those of the ATCC 29906 strain. Consequently, the migration distance of the 1984 strain was shorter by about 32 mm in 24 h of the swarming process, in comparison to the result obtained for ATCC 29906. It is well known that a number of various factors, such as agar concentration [31], inoculation density and incubation temperature [32], and specific nutrients and environmental cues [13] strongly affect the kinetics of the colony area expansion and its appearance. However, it is not an explanation for differences in spreading rates of the 1984 and ATCC 29906 strains incubated in the same conditions. Another possible cause could be a twofold extension of the lag phase of the *P. mirabilis* 1984 strain. Some reports indicate that the lag phase is needed for the excretion of biosurfactants which facilitate microbial motility by lowering the surface tension [33]. Among available papers, only Kannan et al. [34] described *P. mirabilis* DMTMMK1 as a biosurfactant producer. However, preliminary studies did not detect the surfactants’ presence in the swarm colony of either the 1984 or ATCC 29906 strain. On the other hand, delayed differentiation of short-form rods to swarmer cells could be associated with impaired putrescine secretion. Sturgill and Rather [35] indicated that the *P. mirabilis* PM437 strain with a disrupted *speB* gene (encoding putrescine) was featured by a two-hour elongated lag phase, incapability of effective migration across agar surfaces, and formation of very closely spaced swarming rings. However, confirmation of this hypothesis would require supplementation with exogenous putrescine or carrying out research on the 1984 and ATCC 29906 strains at the genetic level. Another cause of varied expansion rates of the *P. mirabilis* 1984 and ATCC 29906 swarm colonies could be differentiated surface density of flagella. Tuson et al. [36] proved that cell length has a small effect on motility but overexpression of FlhD4C2, the major regulator of the flagellar operon in vegetative cells of *P. mirabilis,* increases flagellum density resulting in an increased cell velocity. On the other hand, it is worth mentioning that a delay or a velocity of spreading rods are individual features of each bacterial strain. For the ATCC 29906 and 1984 strains, colony expansion rates were 3.77 mm h^−1^ and 2.45 mm h^−1^, respectively, for *P. mirabilis* BB2000 maximum swarming translocation speed was 7 mm h^−1^ [37], while for *P. mirabilis* PRM1 it reached 1 mm h^−1^ [32], and for *P. mirabilis* HI4320 it was 1.36 mm h^−1^ [14]. Data report that even when the growth kinetics in liquid environments and swarmer cell migrations are quantitatively similar, the minimum length of the lag phase varies by over 40% among different clinical isolates of *P. mirabilis* [32].

High-magnification microscopy showed that the leading edge of the *P. mirabilis* swarmer population is composed of several stacked cell layers, while the population inside the swarming ring forms a monolayer. Swarmer cells translocate from the prior terrace into the newly formed terrace as increasingly thicker populations [32]. In general, the edge of the active swarm front is smooth, while the edge of the newly formed consolidation ring generates finger-like projections [14,36,38]. However, actively swarming colonies of the *P. mirabilis* ATCC 29906 and 1984 strains had a smooth and a “fingered” appearance edge, respectively, and both were in vigorous motion. Only Rauprich et al. [32] indicated that the leading edge of *P. mirabilis* migrating cells was quite ragged in appearance. However, the encroaching swarm fronts of the following strains *Serratia liquefaciens* MG1 [39], *B. subtilis* JCM 32,485 [40], *Bacillus cereus* NCIB 8122 [41], and *Vibrio parahaemolyticus* RIMD 2,210,633 [42] also resembled finger-like structures migrating outward towards the colony border.

The ability of bacteria to control the biophysical properties of their membrane phospholipids enables them to survive under various physical conditions. Moreover, an adjustment in composition of bacterial membranes, i.e., homoviscous adaptation, is interpreted as a mechanism of minimizing energy expenditure and optimizing growth just through modifications of the phospholipid bilayer permeability [22]. Therefore, we com-pared morphologically differentiated forms of the *P. mirabilis* rods in terms of their phospholipid and fatty acid profiles to associate phenotypic changes induced by swarming motility with modifications of these cellular components. Firstly, our results showed that proportions between phosphatidylethanolamines and phosphatidylglycerols were unchanged when the *P. mirabilis* rods were swarming. The same results had been previously described for the *P. mirabilis* P11 [17] and *Paenibacillus polymyxa* ATCC 842 strains [43]. Distribution of phospholipids across the inner membrane of Gram-negative bacteria may be relevant. Bogdanov et al. [44] demonstrated that distribution of PEs was 75% (cytoplasmic leaflet) to 25% (periplasmic leaflet) in rod-shaped cells and quite opposite in elongated *E. coli* cells. Unfortunately, the UHPLC–MS/MS profiling of the lipid composition of whole cells did not provide information on the distribution of these membrane molecules. A dynamic and shape-dependent arrangement of PEs in the inner membrane of Gram-negative bacteria is an interesting finding which suggests that PE distribution facilitates bacterial shape changes or is their consequence. Furthermore, the authors suggested that PE asymmetry is controlled metabolically to balance the synthesis and translocation of the lipid molecules which is supposed to ensure the capacity of envelope growth and adaptation of chemical and physical properties of the bilayer [44]. Despite the constant PG/PE ratio, the 23 phospholipid species showed significantly different levels during the formation of the *P. mirabilis* 1984 and ATCC 29906 swarmers. It was confirmed that drastic modifications in the morphology of the *P. mirabilis* rods were directly related to the deprivation of the long-form cells of PE 33:1 and PG 31:2 and their enrichment with PE 32:1, PE 34:1, PE 34:2, PG 30:2, PG 32:1, and PG 34:1, compared to the short-form cells. On the other hand, the DBI values, indicating the level of PL unsaturation, remained constant for the bacterial rods, independently of their morphotype. These results suggested that the changes in the cells phenotype occurring during swarming motility did not concern the saturation of the phospholipid acyl chains of *P. mirabilis* rods. According to Navas et al. [45], the two-dimensional organization in the PE/PG membranes appears to be dominated by headgroup interactions and to be largely independent of the hydrocarbon chain length or degree of saturation. This is a probable explanation of the constant PG/PE and DBI values, whose modifications caused by *P. mirabilis* rods elongation would be potentially unnecessary.

Bacteria modify the acyl chains of their membrane phospholipids in response to changing environmental conditions. The first of these modifications is *cis*→*trans* isomerization. When the phospholipid *cis*–*trans* isomerase replaces the *cis* conformation of the double bond with the *trans* conformation, *trans* FAs gain properties similar to those for saturated FAs creating membranes with higher transition temperatures [22,46]. The *cis* to *trans* isomerization of FAs, well described for *Pseudomonas putida* and the marine psychrophilic *Vibrio*, seems to be an adaptation of the lipid composition to higher temperatures [46]. This adjustment in the membrane composition does not seem to be useful during the formation of *P. mirabilis* swarmers. Both short and long forms of the ATCC 29906 and 1984 rods contain only *cis* fatty acids. Another modification is cyclopropanation of double bonds. When the cyclopropane fatty acid synthase converts *cis* double bonds of unsaturated fatty acyl chains to cyclopropane rings by transfer of a methylene moiety from S-adenosyl-L-methionine, newly formed *cyclic* FAs stabilize the membranes by increased ordering of the chains, without the limitation of membrane fluidity. This modification, noticed for *E. coli* and *Salmonella enterica* is considered to be crucial for the survival of acid shock [47]. Our results showed decreased levels of *cy* C17:0 in the swarmer cells of the ATCC 29906 and 1984 strains. Similar results were observed for swarmers of *Caulobacter crescentus* [48] and *Pseudomonas aeruginosa* [49]. Limitation of the cyclopropanation reaction seems likely to be important for the differentiation process of *P. mirabilis* elongated cells. The next modification is changing branched-chain fatty acids from *iso* to *anteiso* form, which leads to altering the position of the methyl group on the acyl-chain. The *anteiso* fatty acids promote a higher membrane fluidity than the *iso* fatty acids, due to a dismissed distance of the methyl branch from the FA end. Such adjustment in the membrane FA branching, described for *Listeria monocytogenes*, is regarded as a membrane response to low temperature and pH stress [22]. This adaptation of membrane composition does not seem to be useful when *P. mirabilis* rods undergo the swarming process. In the present study, it was shown that both short-form and long-form rods of the ATCC 29906 and 1984 strains were devoid of *anteiso* fatty acids, whereas Lai et al. [50] reported an increased level of *anteiso* C15:0 in the swarmer cells of the *Serratia marcescens* CH-1. The last modification worth mentioning is a change in the saturation degree of fatty acids. The phospholipid acyl desaturase introduces a *cis* double bond into fatty acids, causing flexion in the chain, which leads to a reduction in transition temperatures and elevation of bilayers permeability. Such regulation of acyl chains packing, described for *Bacillus* spp. and *Pseudomonas* spp., is considered to be necessary for a suitable bacterial reaction to abrupt temperature change [22]. Lack of dependences between the levels of saturated and monounsaturated FAs, as well as between the contents of monounsaturated and double unsaturated FAs containing the same number of carbon atoms in the acyl chains, seems to exclude the activity of desaturases during morphological changes occurring in the swarming rods of the *P. mirabilis* ATCC 29906 and 1984 strains. Gué et al. [18] noticed a reduction in the C16:1 level at the benefit of longer C18 chains of FAs with various degrees of unsaturation in swarmer cells of *P. mirabilis* WT19. However, proportions between the levels of C18:0 and C18:1, as well C18:1 and C18:2, did not indicate the action of desaturases during the swarming of the WT19 strain. Ghorbal et al. [49] showed a significant increase in SFAs accompanied by a significant decrease in UFAs when *P. aeruginosa* PAO1 cells were undergoing the swarming process. A higher level of C18:0 associated with a lower level of C18:1 in the PAO1 swarmers may point to a reduced Δ-9-desaturase activity during cell differentiation. The activity of this enzyme in microorganisms belonging to *Pseudomonas* sp. had been discovered earlier by Garba et al. [51]. On the other hand, Lai et al. [50] presented an increased content of C18:1 in the *S. marcescens* CH-1 swarmer cells but without a simultaneous decline in the C18:0 level, while Chow and Schmidt [48] noticed a simultaneous increase in C16:1 and a decrease in C16:0 amounts when *C. crescentus* cells were swarming. The latter may suggest an enhanced activity of Δ-9-desaturase creating palmitoleic acid based on palmitic acid. So far, this enzyme has not been described in the representatives of *Caulobacter*.

Affinity between lipids may lead to the formation of lipid rafts which stabilize membrane physical properties over varying temperatures and other environmental conditions [28]. One of the tools currently applied to study lipid rafts uses fluorescent molecular probes. The most common are lipophilic fluorescent probes which partition specifically into liquid ordered or liquid disordered phases [30]. In order to search for lipid rafts in the *P. mirabilis* rods morphologically adapted for the swarming phenomenon, we used the energy transfer from the electronic excited donor chromophore (pyrene PE) to the acceptor chromophore (rhod PE). Importantly, previous studies had shown only little effect of the FRET probes presence on domain formation [52]. Differentiation of the *P. mirabilis* cells occurring during the swarming process led to a decrease in F/Fo pointing to the enhancement of the FRET efficiency in the elongated rods of 1984 and ATCC 29906 strains. Melting of rafts taking place during the bacterial rods elongation resulted in an increase in the disordered phase area and spreading of donor and acceptor fluorophores in the membrane area. It corresponded to the homogeneity of lipid bilayers lacking ordered domains. A different result was obtained for the short-form rods of *P. mirabilis*. Weak FRET (an efficient donor quenching) caused by a strong partition of the acceptor chromophore into liquid-disordered domains corresponded to strong membrane ordering of the *P. mirabilis* short cells.

The presence of lipid rafts was noticed in both Gram-negative and Gram-positive bacteria [28,29]. Formation of ordered-domains in *Borrelia burgdorferi* B31 turned out to be similar to the process occurring in eukaryotic cells but with the participation of saturated phosphatidylcholines instead of sphingolipids, crucial components of rafts in eukaryotes [53]. On the other hand, phosphatidylethanolamines behaving similarly to unsaturated PLs were not able to form and stabilize an ordered domain formed spontaneously in the *Helicobacter pylori* membrane lipid extracts [54]. According to the present knowledge, lipids with unsaturated acyl chains are characterized by a decreased ability to create ordered domains relative to lipids containing saturated acyl chains [55]. However, the most notable difference in the phospholipid profiles of the short- and long-form cells of the *P. mirabilis* was a drastic reduction in the PE 33:1 and PG 31:2 total content (~32%) in the first ones. This may indicate an involvement of PE 33:1 and PG 31:2 in the formation of ordered domains in the ATCC 29906 and 1984 short-form rods. Bramkamp and Lopez [56] paid attention to membrane hopanoids in the Gram-negative bacterium *Rhodopseudomonas palustris,* which divides asymmetrically into a mother cell and a swarmer cell. These molecules, similar structurally to sterols, were necessary for proper cell division. Lack of hopanoids led to the formation of cells connected by their cell wall, forming long filaments. Moreover, it was noted that this sterole-like molecule could replace cholesterol in membranes, as well as induce phase separation, which makes it probable that hopanoids are involved in the assembly of lipid rafts, similarly to cholesterol [56]. Research on the lipid rafts in cells undergoing the differentiation process is rather a novelty, hence there is little available information. Mileykovskaya and Dowhan [29] compared wild-type of *E. coli* E614 and its filamentous mutant devoid of membrane PEs with an inhibited cell division process. The authors discovered the cardiolipin-enriched domains in both the filamentous mutant and the septal and cell poles regions of wild-type *E. coli*. Interestingly, in the bacterial envelope there were sites with a higher permeability for used fluorophore, which resulted in different staining of the filamentous mutant and wild-type cells [29]. We obtained similar results. Confocal microscope photographs of Rhod PE-stained membranes visualized a strong fluorescence intensity of the whole *P. mirabilis* swarmer cells in contrast to a weak membrane fluorescence of short-form bacterial rods. A rapid intracellular entry of chromophore could be explained by the lack of membrane tightness of the elongated cells for Rhod PE molecules. Gué et al. [18] also observed an increased membrane fluidity in the swarmer cells of *P. mirabilis* WT19, compared to the vegetative ones. Ghorbal et al. [57] suggested that the cellular fatty acid profile is strongly correlated to the swarming phenotype of *P. aeruginosa* PAO1. Furthermore, Lai et al. [50] hypothesized that the conserved mechanism of swarming in Gram-negative bacteria involves the control of membrane fluidity.

Freeze-fracture electron microscopy and spin-label electron paramagnetic resonance allowed for demonstrating that the mobility of the spin label of the outer membrane of *P. mirabilis* P11 swarmer cells was higher than that of the outer membrane of the non-swarming short cells, which showed a typical rigid profile [58]. It suggested physical reorganization of the outer membrane of swarmer cells associated with an increase in the flagella number per area unit and LPS rearrangement within the membrane, resulting in the exposure of phospholipid regions to the external environment and then altered permeability [17]. Spin labels are usually less rigid in the inner membrane, where the regions of the lipid bilayer enable a rotation of the lipid polar heads and flexing of the nonpolar tails [58]. Molecular relaxations in the acyl region regulate diffusion through the viscosity and elastic properties of the bilayer, such as the bending modulus through the area compressibility [28]. Auer et al. [59] proved that the lengthening of *P. mirabilis* HI4320 cells was accompanied by a large increase in flexibility. The cell stiffness (bending rigidity) was lowered from 1.4 × 10^−20^ N m^−2^ to 5.5 × 10^−22^ N m^−2^ during rods differentiation. The authors found that enhanced flexibility of *P. mirabilis* swarmer cells was caused by modifications in the composition and thickness of the peptidoglycan layer in the walls of their rods [59].

## 4. Materials and Methods

### 4.1. Bacterial Strains

Two *P. mirabilis* strains capable of swarming motility were investigated in this study. The first one, the clinical strain named 1984, came from the strain’s collection of the Department of Biology of Bacteria (University of Lodz, Lodz, Poland). The second, ATCC 29906, which was a reference strain, was purchased from the American Type Culture Collection (Manassas, VA, USA). These bacteria had been described in our previous papers [60,61].

### 4.2. Swarm Plate Assay

The *P. mirabilis* strains were cultured in TSB medium (BioMaxima S.A., Lublin, Poland) at 37 °C for 18 h. Swarming motility was assessed by spotting 5 µL of the late-logarithmic-phase bacterial culture, adjusted to an optical density of 5 on the McFarland scale, onto the center of an LB swarm plate containing 1.5% agar (BioMaxima S.A., Lublin, Poland) followed by incubation at 37 °C. The diameter of the swarm colony was measured at two-hour intervals for 24 h by using a caliper (Schut Geometrical Metrology, Giethoorn, The Netherlands).

### 4.3. Phase-Contrast Microscopy Imaging

An eclipse TE2000-S Inverted Microscope (Nikon, Tokyo, Japan) equipped with an air condenser (Nikon LWD, numerical aperture (NA) 0.52) and a 20× phase contrast lens (Nikon, LWD, Ph1, NA 0.40) was applied for imaging the *P. mirabilis* rods spreading over the agar surface. After 18 h of the swarm plate assay (Section 4.2) duration, part of the agar containing the outermost edge of the swarm colony was cut with a scalpel (Swann-Morton, Sheffield, England) and transferred onto a microscopic slide (Menzel Gläser, Braunschweig, Germany). Initially, the sample was illuminated through a 4× objective (NA = 0.10) to capture a wide field. Subsequently, the objective LWD 20× was used for the presentation of the *P. mirabilis* colony movement in 4-min intervals. 

Phase-contrast microscopy was also applied for imaging the elongated *P. mirabilis* rods. After 18 h of the swarm plate assay (Section 4.2) duration, the bacterial cells from the outermost edge of the swarm colony were imprinted to microscopic slides (Menzel Gläser, Braunschweig, Germany). Observations were carried out using a 60× Plan Fluor objective (Nikon, ELWD, Ph2, NA 0.70).

### 4.4. Preparing Bacteria for Further Research

After 18 h of the swarm plate assay (Section 4.2) duration, the *P. mirabilis* cells from two zones, i.e., consolidation and the outermost edge of the swarm colony, were collected from the agar plate surface using sterile swabs (PROFILAB, Warsaw, Poland) and then suspended in 1 mL of sterile distilled water or PBS solution. Optical densities of the cell suspensions were adjusted spectrophotometrically to 0.4 (Multiskan EX, LabSystem, Cracow, Poland) at λ = 550 nm. The short- and long-form cells prepared in this way were used for further experiments. Each of them was preceded by the verification of the microscopic morphology of gram-stained bacterial forms.

### 4.5. Extraction and Quantification of Bacterial Phospholipids

The *P. mirabilis* lipids were isolated in accordance with our gently modified method described previously [62]. The bacterial cells obtained according to the description in Section 4.4 were centrifuged for 15 min at 12,000 rpm (Sigma 1-15 Centrifuge; SIGMA Laborzentrifugen GmbH, Osterode, Germany). Firstly, 1 mL of the mixture (2:1 *v*/*v*) of chloroform-methanol (Chempur, Piekary Slaskie, Poland) and glass beads (Ø 1 mm) (Merck, Darmstadt, Germany) were added into an Eppendorf containing the cells washed in distilled water. Rods disintegration and a simultaneous transfer of the lipids to the organic phase were performed using a ball mill (Retsch MM 400, Haan, Germany) for 5 min at 30 Hz. Then, 100 µL of 0.85% NaCl was added to the homogenate devoid of glass beads. After quick mixing on the vortex, the sample was centrifuged (Sigma 1-15 Centrifuge; SIGMA Labor-zentrifugen GmbH, Osterode, Germany) for 5 min at 2000 rpm. A bottom layer was collected and dried.

Lipid extracts were dissolved in ultrapure methanol (J.T. Baker Chemical Company, Deventer, the Netherlands) and then analyzed using an ExionLC AC UHPLC system (Sciex, Framingham, MA, USA) coupled with a 4500 QTRAP mass spectrometer (Sciex, Framingham, MA, USA). Ion source, column specification, gradient and flow of solvents, and other parameters had been presented in more detail in our earlier paper [61]. To acquire accurate and precise quantification results, the internal standards PE 14:0/14:0 and PG 14:0/14:0 (Merck, Darmstadt, Germany) were applied. For phospholipid analysis, an information-dependent acquisition method (precursor ion or multiple reaction monitoring enhanced product ion) was also employed. The mass spectra of PGs and PEs were acquired in the range of *m*/*z* 100–1500 and exhibited the ions corresponding to the deprotonated molecules [M − H]^−^.

Values of the double bond index referring to the degree of unsaturation of phospholipid tails were calculated in accordance with Su et al. [63].

### 4.6. Isolation and Identification of Bacterial Fatty Acids

The *P. mirabilis* fatty acids contained in the extract prepared according to the description in Section 4.5 were released and derivatized to form fatty acid methyl esters (FAMEs) using the previously described procedure [61]. In short, methanolic HCl (8%) and toluene (Avantor Performance Materials Poland S.A, Gliwice, Poland) were added to the lipid extracts and then the solutions were heated at 45 °C for at least 18 h (Eppendorf Thermomixer R with Eppendorf 2 ml Thermoblock; Eppendorf, Hamburg, Germany). After derivatization, the samples were cooled and the FAMEs were extracted with ultrapure hexane (J.T. Baker Chemical Company, Deventer, the Netherlands) for further GC–MS analyses. 

The upper layers of the solutions, containing methylated bacterial fatty acids, were placed into the inserts of chromatographic vials (Supelco Inc., Bellefonte, PA, USA) and then analyzed using an Agilent Model 7890 gas chromatograph equipped with a 5975C mass detector (Santa Clara, CA, USA). Settings of the injection port, temperature gradient of the capillary column, helium flow, and other method details were provided in our previous work [61]. The reference standards of bacterial fatty acid methyl esters (Matreya LLC, State College, PA, USA) were applied for the quantitative method.

### 4.7. Förster Resonance Energy Transfer Technique

Segregation of lipids in the short- and long-form rods of *P. mirabilis* was detected using a FRET technique in accordance with the modified method of Huang et al. [54]. Two membrane probes labeled at the polar head group constituting a donor-acceptor pair were applied. 1,2-dipalmitoyl-sn-glycero-3-phosphoethanolamine-N-(1-pyrenesulfonyl)—pyrene PE was a donor fluorophore, while 1,2-dioleoyl-sn-glycero-3-phosphoethanolamine-N-(lissaminerhodamine B sulfonyl)—rhod PE (Avanti Polar Lipids, Alabaster, AL, USA) was an acceptor fluorophore.

Bacterial cells suspended in PBS solution (prepared according to the description in Section 4.4) were portioned by 200 µL into the wells of a black 96-well plate (Thermo Fisher Scientific, Roskilde, Denmark) obtaining ~6.5 × 10^6^ cells per well, and then labeled with membrane probes. The F wells contained the FRET donor (to a final concentration of 0.02 µg mL^−1^) and the FRET acceptor (to a final concentration of 1 µg mL^−1^). The Fo wells contained only the donor. Backgrounds for the F wells (containing unlabeled cells and the acceptor, but no donor) and for the Fo wells (containing only unlabeled cells) were also prepared. The plate was incubated in the dark at room temperature for 1 h before fluorescence measurements. By applying the same conditions of the experiments, paying special attention to the age (18 h) and density of the analyzed bacterial cells (~6.5 × 10^6^ cells), the concentration of fluorophores, and the labeling time, the impact of the above factors on the stability of the experimental FRET values, as well as on the repeatability of the obtained results, was minimalized.

Fluorescence intensity was measured in the Laboratory of Microscopic Imaging and Specialized Biological Techniques, Faculty of Biology and Environmental Protection, University of Lodz, Poland using a Spectramax i3 Multi-Mode Microplate Reader (Syngen Biotech, Wroclaw, Poland) with the following parameters: λex = 350 nm and λem = 379 nm, 6 flashes on read, a linear shaking before read for 5 s. The F/Fo values were calculated after the subtraction of backgrounds. The values of fractions of energy transfer were calculated with the following formula 1-F/Fo.

### 4.8. Confocal Microscopy Imaging

The *P. mirabilis* rods obtained according to the description in Section 4.4 were transferred into a 24-well black plate with a transparent glass bottom (Greiner Bio-One GmbH, Kremsmünster, Austria) and then labeled with Rhod PE (Avanti Polar Lipids, Alabaster, AL, USA) for 18 h, allowing the cells to adhere to the glass surface. Labeled cells were imaged using a DMI 6000 CS inverted microscope with the TCS SP8 confocal system, operated by the LAS 2.0.215022 software (Leica Microsystems, Wetzlar, Germany) in the Laboratory of Microscopic Imaging and Specialized Biological Techniques, Faculty of Biology and Environmental Protection, University of Lodz, Poland. The observations were made using an HC PLAPO CS2 100×/1.40 oil immersion objective. The 560 nm supercontinuum white light laser (WLL) (10% intensity) was used to excite the fluorophore-stained membranes. A fluorescence emission was collected in the range of 570–699 nm by a conventional detector photomultiplier tube (PMT) or by a hybrid detector (HyD) working in bright R mode. To enhance the contrast, a green channel was applied. Confocal scans were performed at a speed of 600 Hz, zoom from 1.8 to 3.61, a pinhole 151.5 µm, and a line average set at 7. Image logical size was 512 × 512.

### 4.9. Statistical and Other Analyses 

The presented data are shown as the mean value and standard deviation of three separate analyses. The significance of results was determined by Fisher’s tests and two-sample T tests assuming that equal or unequal variances were appropriate. All p-values are one tailed at a 95% confidence interval. Analyses were performed by using Excel 2016 (Microsoft Corporation, Redmond, WA, USA), the GraphPad Prism 9.1.0 software (GraphPad, San Diego, CA, USA), and the Analyst v1.6.3 software (Sciex, Framingham, MA, USA).

## 5. Conclusions

Our studies clearly demonstrate that swarming motility regulates the lipid composition and organization in *P. mirabilis* rods. We have proved that drastic modifications occurring during the formation of the long-form cells of uropathogens are directly related to changes in their phospholipid profile, while showing weak relation with the alterations in their fatty acid composition. We have indicated some phospholipid species as probable participants in the formation and stabilization of lipid rafts present in the short-form rods of *P. mirabilis*. Melting of these domains during the rod elongation has been found to ensure homogeneity of the lipid bilayers and enhance their permeability for macromolecules.

In this study, we have been able to gain important insights into the relevance of the lipid adjustment, a factor involved in phenotypic modifications induced in *P. mirabilis* rods during swarming motility. However, this preliminary report requires expansion in order to search for other potential lipid virulence factors associated with the elongated morphotype of swarming rods that potentially contribute to UTIs development.

## Figures and Tables

**Table 1:** Relative abundances (%) of phospholipids isolated from the swarmer and non-swarmer cells of the *P. mirabilis* strains.

PL Species	*P. mirabilis* ATCC 29906 Cells			*P. mirabilis* 1984 Cells		
	Short-Form	Long-Form		Short-Form	Long-Form	
PE 30:0	4.13 ± 0.4	3.84 ± 0.4	ns	4.02 ± 0.2	3.64 ± 0.6	ns
PE 30:1	1.44 ± 0.1	1.84 ± 0.2	ns	0.88 ± 0.0	1.31 ± 0.2	*
PE 31:0	2.35 ± 0.1	0.18 ± 0.2	***	2.12 ± 0.0	0.51 ± 0.5	**
PE 32:0	2.90 ± 0.1	4.59 ± 0.5	*	3.79 ± 0.2	5.01 ± 1.7	ns
PE 32:1	14.6 ± 0.3	20.6 ± 0.4	*	13.1 ± 0.2	21.1 ± 0.4	**
PE 32:2	0.33 ± 0.1	0.35 ± 0.3	ns	0.25 ± 0.0	0.86 ± 0.2	**
PE 33:0	2.74 ± 0.1	0.11 ± 0.1	***	2.80 ± 0.1	0.09 ± 0.0	***
PE 33:1	23.5 ± 0.2	1.49 ± 0.2	***	23.9 ± 0.5	0.63 ± 0.8	***
PE 34:0	0.85 ± 0.1	1.17 ± 0.1	*	1.05 ± 0.1	1.46 ± 0.2	ns
PE 34:1	7.28 ± 0.7	12.4 ± 1.2	**	9.61 ± 0.5	13.0 ± 1.4	*
PE 34:2	4.78 ± 0.8	11.3 ± 0.7	**	2.20 ± 0.6	13.2 ± 0.5	***
PE 36:0	0.15 ± 0.0	0.94 ± 0.3	**	0.10 ± 0.0	0.65 ± 0.3	***
PE 36:2	0.81 ± 0.2	1.30 ± 0.2	**	0.82 ± 0.1	0.85 ± 0.5	ns
PE 37:2	1.54 ± 0.1	2.34 ± 0.2	**	1.39 ± 0.0	1.95 ± 0.2	**
PE 38:2	2.89 ± 0.3	0.07 ± 0.1	***	3.20 ± 0.0	0.08 ± 0.1	***
PG 28:1	1.59 ± 0.1	1.17 ± 0.4	ns	1.66 ± 0.1	1.24 ± 0.3	*
PG 29:2	0.91 ± 0.6	0.10 ± 0.7	ns	0.87 ± 0.0	0.37 ± 0.5	ns
PG 30:0	0.36 ± 0.1	0.98 ± 0.4	*	0.24 ± 0.0	1.02 ± 0.4	**
PG 30:1	0.73 ± 0.1	1.08 ± 0.4	ns	1.23 ± 0.1	1.51 ± 0.6	ns
PG 30:2	5.09 ± 0.3	7.92 ± 0.2	***	4.99 ± 0.6	7.77 ± 0.5	***
PG 31:2	11.0 ± 0.6	0.81 ± 0.2	***	10.9 ± 1.1	0.95 ± 0.4	***
PG 32:1	2.11 ± 0.2	9.98 ± 1.5	***	1.74 ± 0.1	8.14 ± 0.1	***
PG 32:2	2.96 ± 0.3	5.30 ± 0.2	*	3.61 ± 0.4	5.32 ± 0.6	*
PG 33:1	2.94 ± 0.3	0.59 ± 0.3	***	2.84 ± 0.3	0.65 ± 0.3	***
PG 34:0	0.22 ± 0.0	0.85 ± 0.2	***	0.27 ± 0.0	0.89 ± 0.2	**
PG 34:1	1.86 ± 0.1	8.65 ± 1.2	***	2.50 ± 0.1	7.80 ± 1.3	***
PG/PE ratio	0.43 ± 0.0	0.59 ± 0.2	ns	0.45 ± 0.0	0.54 ± 0.1	ns
DBI	1.17 ± 0.0	1.17 ± 0.1	ns	1.14 ± 0.0	1.18 ± 0.2	ns

Values represent mean percentages of the phospholipid pool. The error values equal the standard deviations. PG: phosphatidylglycerol, PE: phosphatidylethanolamine, DBI: double bond index. Statistical significance is indicated as * (*p*-value ≤ 0.05), ** (*p*-value ≤ 0.01), *** (*p*-value ≤ 0.001), and ns means not significant (*p*-value > 0.05).

**Table 2:** Relative abundances (%) of fatty acids determined in the swarmer and non-swarmer cells of the *P. mirabilis* strains.

FAs	*P. mirabilis* ATCC 29906 Cells			*P. mirabilis* 1984 Cells		
	Short-Form	Long-Form		Short-Form	Long-Form	
C14:0	1.95 ± 0.3	2.04 ± 0.3	ns	1.95 ± 0.2	1.77 ± 0.1	ns
C15:0	0.88 ± 0.8	1.75 ± 0.2	ns	1.84 ± 0.1	1.47 ± 0.2	ns
*iso*-C16:0	1.64 ± 0.3	1.83 ± 0.2	ns	1.34 ± 0.3	1.48 ± 0.2	ns
C16:0	36.5 ± 1.1	36.5 ± 4.2	ns	39.0 ± 2.8	36.8 ± 0.7	ns
*cis* C16:1	2.37 ± 0.4	2.67 ± 0.5	ns	2.43 ± 0.1	2.05 ± 0.4	ns
C17:0	1.68 ± 0.3	1.93 ± 0.2	ns	1.63 ± 0.2	1.63 ± 0.2	ns
*cy* C17:0	3.01 ± 0.8	1.76 ± 0.3	*	3.89 ± 0.2	1.46 ± 0.2	***
C18:0	44.0 ± 1.6	43.5 ± 4.3	ns	39.9 ± 3.5	46.0 ± 1.9	*
*cis* C18:1	2.52 ± 0.5	2.45 ± 0.3	ns	2.90 ± 0.4	2.18 ± 0.2	ns
C19:0	1.65 ± 0.3	1.19 ± 1.1	ns	1.39 ± 0.2	1.58 ± 0.2	ns
*cy* C19:0	1.90 ± 0.4	1.99 ± 0.3	ns	2.13 ± 0.1	1.66 ± 0.2	**
C20:0	1.98 ± 0.4	2.32 ± 0.3	ns	1.64 ± 0.3	1.96 ± 0.2	ns
C16/C18	0.87 ± 0.0	0.89 ± 0.2	ns	1.00 ± 0.2	0.84 ± 0.0	ns
SFA	90.2 ± 1.9	91.1 ± 1.2	ns	88.7 ± 0.7	92.6 ± 0.8	***
UFA	4.89 ± 0.9	5.12 ± 0.7	ns	5.33 ± 0.5	4.24 ± 0.5	*
UFA/SFA	0.05 ± 0.0	0.06 ± 0.0	ns	0.06 ± 0.0	0.05 ± 0.0	ns
CFA	4.91 ± 0.1	3.75 ± 0.5	ns	6.02 ± 0.2	3.12 ± 0.3	***
CFA/UFA	1.00 ± 0.0	0.73 ± 0.0	***	1.13 ± 0.1	0.74 ± 0.1	***
straight/branched-chain FAs	9.20 ± 2.2	10.3 ± 1.6	ns	7.81 ± 0.5	12.6 ± 1.5	*

Values represent mean percentages of the total fatty acids pool. The error values equal the standard deviations. SFA: saturated fatty acids, UFA: unsaturated fatty acids, CFA: cyclopropane fatty acids, FAs: fatty acids. Statistical significance is indicated as * (*p*-value ≤ 0.05), ** (*p*-value ≤ 0.01), *** (*p*-value ≤ 0.001), and ns means not significant (*p*-value > 0.05).

## Data Availability

Data are contained within the article.
